# Pharmacokinetics and Pharmacodynamics of Recombinant Human EPO-Fc Fusion Protein *In Vivo*

**DOI:** 10.1371/journal.pone.0072673

**Published:** 2013-08-19

**Authors:** Xunlong Shi, Jianjun Yang, Haiyan Zhu, Li Ye, Meiqing Feng, Jiyang Li, Hai Huang, Qun Tao, Dan Ye, Lee-Hwei K. Sun, Bill N. C. Sun, Cecily R. Y. Sun, Guizhen Han, Yuanyuan Liu, Minghui Yao, Pei Zhou, Dianwen Ju

**Affiliations:** 1 Department of Biosynthesis, School of Pharmacy, Fudan University, Shanghai, China; 2 Shanghai Meiye Biotech Institute, Shanghai, China; 3 PharMab (Shanghai), Inc., Shanghai, China; 4 Department of Pharmacology, School of Medicine, Fudan University, Shanghai, China; IIT Research Institute, United States of America

## Abstract

In this study, the *in vivo* pharmacokinetics and pharmacodynamics of a novel recombinant human erythropoietin (rhEPO) Fc fusion protein, rhEPO-Fc, were studied in both rodents and rhesus monkeys. Animal models of anemia induced by irradiation, cyclophosphamide and partial renal ablation were used to evaluate therapeutic effects of rhEPO-Fc. We have demonstrated that serum half-life of rhEPO-Fc was 29.5 to 38.9 h at doses of 8, 25, 80 µg/kg in rhesus monkeys and 35.5 to 43.5 h at doses of 16, 50, 160 µg/kg in rats. In anemia animal models, rhEPO-Fc dose-dependently (7.5–30.0 µg/kg in mice, 5.4–21.4 µg/kg in rats and 5.0–10.0 µg/kg in rhesus monkeys) increased reticulocyte level, followed by an increase of RBC count, hemoglobin and hematocrit levels. At reduced intervention frequency of weekly treatments, rhEPO-Fc showed similar hematopoietic effects as compared with rhEPO given three times a week. These results indicated that rhEPO-Fc could potentially be used in treatment of anemia and warrants future clinical trials.

## Introduction

Erythropoietin (EPO) is a glycoprotein that stimulates the production of erythrocytes [1], [2]. It promotes proliferation, differentiation and maturation of erythroid progenitor cells, and inhibits their apoptosis [3]. In clinical practice, recombinant human EPO (rhEPO) has been used in treatment for anemia associated with chronic renal failure [4], cancer chemotherapy [5], HIV infection [6], and a number of other pathological conditions [7], [8].

Since initial clinical usage of rhEPO-α in the 1980s, clinicians quickly recognized the need of frequent administration as one of major drawbacks of the drug. This imposes a burden on both patients and health care providers, as *in vivo* half-lives of rhEPO-α and rhEPO-β administered intravenously or subcutaneously in humans are only about 8.5 and 17 hours respectively [9], [10]. Thus, there has been a longstanding need to develop recombinant EPO analogs with longer *in vivo* half-lives.

Attempts have been made to genetically or chemically modify the structure of native EPO protein to either slow down its *in vivo* metabolism or improve its therapeutic properties [11], [12]. To extend the half-life, EPO has been chemically conjugated with other moieties to increase its molecular weight. For instance, polyethylene glycol conjugated (PEGylated) EPO has a much higher molecular weight and is protected from being cleared from circulation and therefore has a longer plasma half-life. However, PEGylation may alter the protein structure resulting in unanticipated changes of function and specificity of EPO moiety [13], [14]. Other strategies have also been reported to increase the molecular weight of EPO, such as linking EPO molecule to a carrier protein (e.g. human albumin), or forming a homo-dimer of two EPO molecules using linking peptides (3- to 17-amino acids) or chemical cross-linkers [15]–[18].

To develop a “controlled release” of EPO, we have previously developed a novel recombinant human EPO fused with an Fc domain from a modified human IgG2 without CDC and ADCC function [19], [20]. In the present report, its pharmacokinetics and pharmacodynamics were studied. The erythropoietic effects were investigated in various rodent as well as nonhuman primate anemia models.

## Materials and Methods

### Materials

Recombinant human EPO (rhEPO, 30.4 kD, 98% purity) was purchased from 3SBio Inc. China. The novel EPO fusion protein, rhEPO-Fc (118 kD, ∼60,000 IU/mg) was supplied by Shanghai Meiye Biotech Institute. Briefly, the plasmid encoding rhEPO-Fc fusion protein, containing human EPO, Fc domain of human IgG2 and 16-amino acid linker, was transfected and expressed in Chinese Hamster Ovary (CHO) cells with a yield of 2.5 g/L. The fusion protein was purified using a Protein-A column and the final purity is more than 95% by SDS-PAGE analysis [19], [20].

### Animals

C57BL/6J mice (20.4 ± 0.3 g, 4 weeks old) and Sprague-Dawley rats (232.8 ± 2.9 g, 8 weeks old) were purchased from Sino-British Sippr/BK Lab. Animal Co. Ltd. Rhesus monkeys (3.9 ± 0.3 kg), for *in vivo* bioactivity study, were from Shanghai Public Health Clinical Center, Shanghai, China, and SPF rhesus monkeys (3.5–5.0 kg) for pharmacokinetics study were from Chengdu Greenhouse Biotech Co. Ltd, Chengdu, China.

Animals were housed under specific pathogen free conditions. All experimental protocols were approved by the Animal Experiment Committee of Fudan University, Shanghai, China.

### Pharmacokinetic study

For single dose pharmacokinetics study, rats (n = 12 per group) were injected subcutaneously with 16, 50, or 160 µg/kg of rhEPO-Fc, and for rhesus monkeys (n = 6 per group), the doses were 8, 25, or 80 µg/kg. Blood samples (0.3 ml) were collected via retro-orbital venous plexus for rats and femoral vein for rhesus monkeys during 0–168 h after rhEPO-Fc administration, and serum levels of rhEPO-Fc were determined.

For repeated dose pharmacokinetics study, rhesus monkeys (n = 6) were injected subcutaneously with rhEPO-Fc at 25 µg/kg weekly for four consecutive weeks. After the 1^st^ and 4^th^ injection, blood samples were collected in various time points till 168 h. The serum concentration of rhEPO-Fc was determined using an ELISA kit from R&D Systems (Minneapolis, MN, USA).

Pharmacokinetics parameters were obtained by fitting the data to an extravascular administration model with a first order adsorption phase and a first order elimination phase.

### Irradiation induced anemia in mice

C57BL/6J mice received a fractionated total body irradiation (TBI) at a dose of 2×4 Gy (5 MeV photons generated by a linear accelerator at a rate of 2.5 Gy/min), on two consecutive days [21]. Two days after irradiation, mice (n = 14 per group) were subcutaneously injected with rhEPO-Fc (7.5, 15.0, 30.0 µg/kg, once a week), control rhEPO (7.5 µg/kg, 3 times per week), or PBS (sterile, endotoxin-free Phosphate Buffer Solution) for a total period of 4 weeks. At indicated time points (0–34 day), red blood cell (RBC), hemoglobin, hematocrit, reticulocyte were determined with an automatic blood analyzer (MEK-8222k, Optoelectronic Industry Co. Ltd, Tokyo, Japan).

### Partial renal ablation induced anemia in rats

Seven days after the right kidney was surgically removed, the cortex of the left kidney was partially resected (A quantity corresponding to 2/3 of the weight of the previously resected right kidney). Sham control rats received identical surgical procedures, but no kidney or kidney tissues were removed [22]. Five days after surgery, rats (n = 16 per group) were subcutaneously injected with rhEPO-Fc (5.4, 10.7, 21.4 µg/kg, given weekly), control rhEPO (5.4 µg/kg, given 3 times a week), or PBS for four weeks.

Blood samples were collected weekly during treatment period, and two weeks after treatment ended. RBC, hemoglobin, hematocrit, reticulocyte and blood urea nitrogen (BUN) were evaluated.

### Cyclophosphamide induced anemia in rhesus monkeys

Cyclophospamide is an alkylating nitrogen mustard which is clinically used to treat numerous types of cancer and certain autoimmune disorders. Bone marrow suppression, including anemia, is one of the major adverse effects [23], [24]. To induce anemia, rhesus monkeys were given intravenously twice a day at a dose of 50 mg/kg cyclophosphamide on two consecutive days. Two days after the second injection, monkeys (n = 4 per group) were subcutaneously injected with rhEPO (2.5 µg/kg, 3 times per week), rhEPO-Fc (5.0, 10.0 µg/kg weekly) or PBS for seven weeks. Blood samples were collected at 0–49 days during treatment period and additional 4 times weekly after treatment ended. RBC, hemoglobin, hematocrit, reticulocyte, blood platelet and leukocyte were determined as described above.

### Statistical Analysis

Pharmacokinetics and pharmacodynamics data were analyzed using one-way ANOVA, and a *p* value ≤0.05 was considered statistically significant.

## Results

### Pharmacokinetics

**Figure 1:**
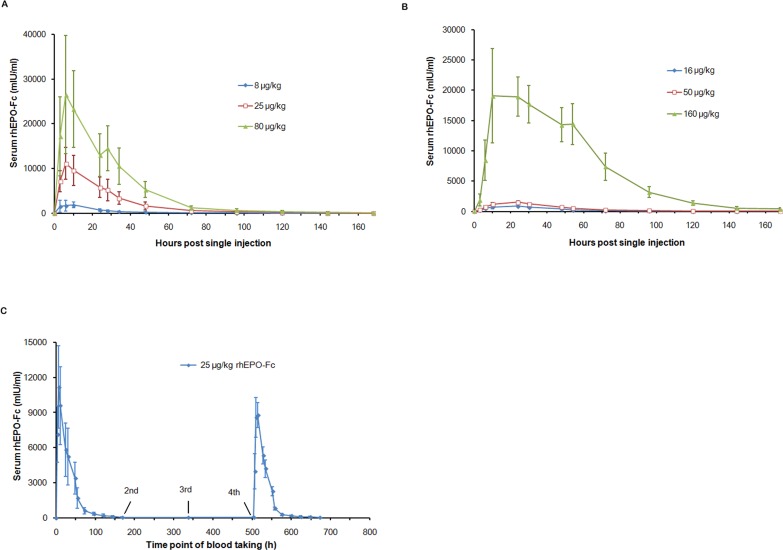
Pharmacokinetics of rhEPO-Fc with single and repeated injections. Mean serum rhEPO-Fc concentration versus time after single subcutaneous injection in rhesus monkeys (Fig. 1A), rats (Fig. 1B) and repeated subcutaneous injections in rhesus monkeys (Fig. 1C) were plotted. Data were mean±SD for six rhesus monkeys or twelve rats per group.

To evaluate pharmacokinetic behavior of the rhEPO-Fc fusion protein *in vivo*, both single and repeated rhEPO-Fc injections were conducted in both rats and rhesus monkeys. Circulating levels of rhEPO-Fc following single injections were measured by ELISA. The mean serum concentration-time curves were shown in Fig. 1A and Fig. 1B for rhesus monkeys and rats, respectively. The pharmacokinetics parameters were summarized in Table 1.

**Table 1:** Pharmacokinetic parameters of rhEPO-Fc following single subcutaneous injection in rhesus monkeys and rats.

Species	Dose	N	Cmax^a^	Tmax^b^	AUC_(0–168)_ c	CL^d^	Half-life
	(µg/kg)		(IU/L)	(h)	(IU/ h·ml)	(ml/h/kg)	(h)
**Rhesus monkey**	8	6	2193.1±902.3	7.5±3.0	56.3±19.0	0.4±0.2	38.9±16.3
	25	6	12186.1±2695.5	8.0±2.2	335.0±81.1	0.2±0.1	29.5±4.2
	80	6	26772.4±13066.6	6.7±1.6	837.0±295.2	0.3±0.1	33.1±14.1
**Rat**	16	12	899.6±141.9	21.7±5.7	39.0±5.9	0.5±0.1	38.6±22.9
	50	12	1522.5±209.1	24.0±0.0	69.2±6.7	0.5±0.3	35.5±8.9
	160	12	22191.4±4422.3	23.0±17.4	1238.0±116.5	0.3±0.3	43.5±32.0

a Maximal drug concentration.

b Time of maximal drug concentration.

c Area under the curve (0–168 h).

d Clearance.

In rhesus monkeys (Fig. 1A and Table 1, the C_max_ and AUC_(0–168 h)_ positively correlated with dosage. With increasing doses of rhEPO-Fc, C_max_ and AUC_(0–168 h)_ correspondingly increased, but T_max_, CL and half-life did not show obvious dose-dependent effects. The serum half-life of rhEPO-Fc was 29.5 to 38.9 h.

In rats (Fig. 1B and Table 1), the similar dose-dependent correlations of C_max_ and AUC_(0–168 h)_ were also observed. The half-life of rhEPO-Fc was 35.5 to 43.5 h.

In rhesus monkeys receiving repeated injections, there was no rhEPO-Fc detected before the 2^nd^, 3^rd^ and 4^th^ injection (Fig. 1C). After the 4^th^ injection, the serum concentration-time curve of rhEPO-Fc did not change significantly, similar to that of the 1^st^ injection. This indicated that the repeated injections did not change the pharmacokinetic behavior of rhEPO-Fc.

### rhEPO-Fc alleviates anemia induced by irradiation

Next, we tested whether rhEPO-Fc have hematopoietic effects in animal anemia models. We first evaluated the effects of rhEPO-Fc on anemic mice induced by total body irradiation (TBI).

**Figure 2:**
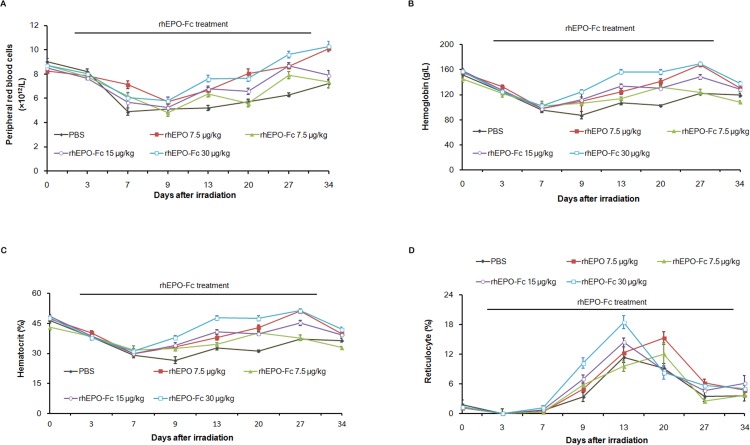
rhEPO-Fc attenuated anemia induced by irradiation in mice. Blood samples were taken at day 0, 3, 7, 9, 13, 20, 27 and 34 to determine RBC (Fig. 2A), hemoglobin (Fig. 2B), hematocrit (Fig. 2C), and reticulocyte (Fig. 2D). Data were presented as Mean±SD.

As shown in Fig. 2A, TBI induced severe anemia in C57BL/6 mice. The mean RBC count of PBS control mice decreased to the lowest level (4.9×10^12^/L, 54% of baseline) on day 7 post irradiation and recovered to 7.2×10^12^/L (80% of baseline ) four weeks later. In mice treated with rhEPO-Fc (7.5, 15.0, 30.0 µg/kg, weekly), rhEPO-Fc treatments attenuated reduction of RBC in a dose-dependent manner. The RBC counts on day 7 were 6.2×10^12^/L, 5.7×10^12^/L, 6.1×10^12^/L in mice treated with rhEPO-Fc at doses of 7.5, 15 and 30.0 µg/kg, decreased to the lowest level on day 9 and then recovered. On day 13 and 27, RBC counts of mice treated with 15 and 30.0 µg/kg rhEPO-Fc were significantly higher than those in PBS control group.

Hemoglobin and hematocrit (Fig. 2B and 2C) were also significantly lowered after irradiation similar to RBC (Fig. 2A). Treatment with rhEPO-Fc also dose-dependently alleviated the reduction of hemoglobin and hematocrit (*p*≤0.05, compared with PBS control). At the weekly dosing of 30.0 µg/kg, rhEPO-Fc exerted similar effects on RBC, hemoglobin and hematocrit, compared with rhEPO (7.5 µg/kg, 3 times a week).

After irradiation, reticulocyte counts of PBS control mice reduced to the lowest level on day 3, then rapidly reached the highest level on day 13 and then decreased gradually. The rhEPO-Fc treatment showed dose-dependent stimulating effects on reticulocytes, which significantly elevated at doses of 15.0, 30.0 µg/kg on day 9 and day 13 as compared with those in rhEPO-treated mice) (*p*≤0.05) (Fig. 2D).

These data demonstrated that rhEPO-Fc dose-dependently attenuated reduction of RBC, hemoglobin and hematocrit, rapidly elevated levels of reticulocyte in anemia induced by TBI in mice.

### rhEPO-Fc alleviates anemia induced by partial renal ablation

**Figure 3:**
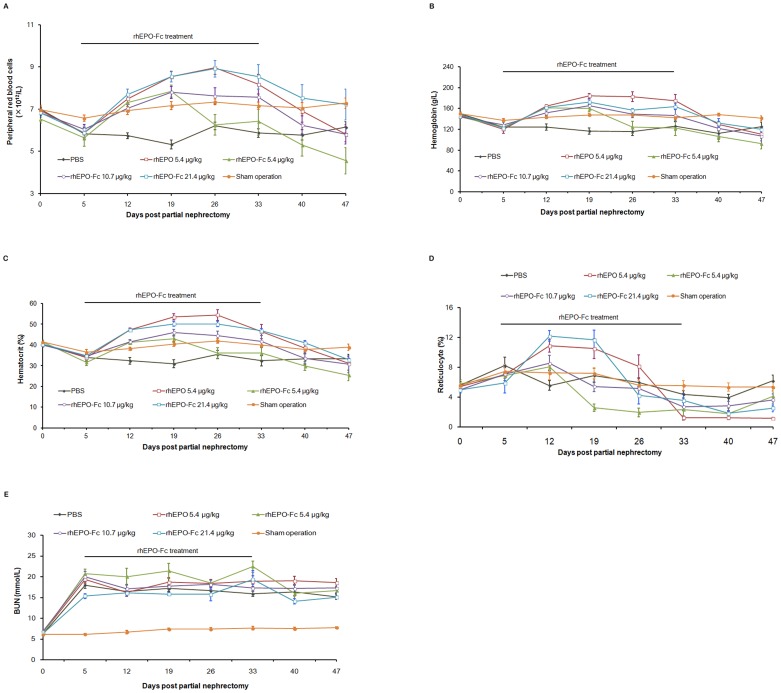
rhEPO-Fc attenuated anemia induced by partial renal ablation in rats. Blood samples were collected at the indicated time points (0–4 week, and additional two weeks after the treatment ended). RBC (Fig. 3A), hemoglobin (Fig. 3B), hematocrit (Fig. 3C), reticulocyte (Fig. 3D) and BUN (Fig. 3E) were shown. Data were presented as Mean±SD.

The erythropoietic effect of rhEPO-Fc was also evaluated in anemia induced by partial renal ablation in rats. The mean RBC count decreased to the lowest level (5.3×10^12^/L, 76% of baseline) on day 19 after partial nephrectomy, and remained at this level throughout the entire experiment (Fig. 3A). However, when treated with rhEPO-Fc (5.4, 10.7, 21.4 µg/kg weekly), on day 19, RBC counts significantly increased (7.9×10^12^/L, 7.8×10^12^/L, 8.5×10^12^/L) in animals treated with all three doses of rhEPO-Fc. In anemic rats treated with rhEPO-Fc at doses of 10.7 and 21.4 µg/kg, the RBC counts were restored to the sham operation levels, significantly higher than those of PBS-treated anemic rats (*p*≤0.05) in the course of treatment. At the end of the treatment, RBC counts of rats treated with rhEPO-Fc (21.4 µg/kg weekly) reached similar levels, as compared with those in mice treated with rhEPO (5.4 µg/kg, 3 times a week).

Similar dose-dependent stimulating effects on hemoglobin and hematocrit were also observed (Fig. 3B and 3C). During rhEPO-Fc therapy with weekly doses of 10.7 and 21.4 µg/kg, hemoglobin and hematocrit levels were restored to the sham operation level. The effects of rhEPO-Fc (21.4 µg/kg) were similar to those of rhEPO treatment (5.4 µg/kg, 3 times a week).

After the partial renal ablation surgery, reticulocytes of PBS-treated rats rapidly increased and changed in fluctuation. At the dose of 21.4 µg/kg, rhEPO-Fc exerted the strongest effect on reticulocyte (243% of baseline at day 12, *p*≤0.05, *vs* PBS), similar to those of rhEPO therapy (5.4 µg/kg, 3 times a week) (Fig. 3D).

The partial renal ablation also resulted in high levels of BUN in the rats and neither rhEPO nor rhEPO-Fc treatment affected BUN (Fig. 3E). Although administration of rhEPO-Fc did not improve impaired kidney function, it effectively corrected anemia induced by partial renal ablation.

### rhEPO-Fc attenuates anemia induced by cyclophosphamide in rhesus monkeys

**Figure 4:**
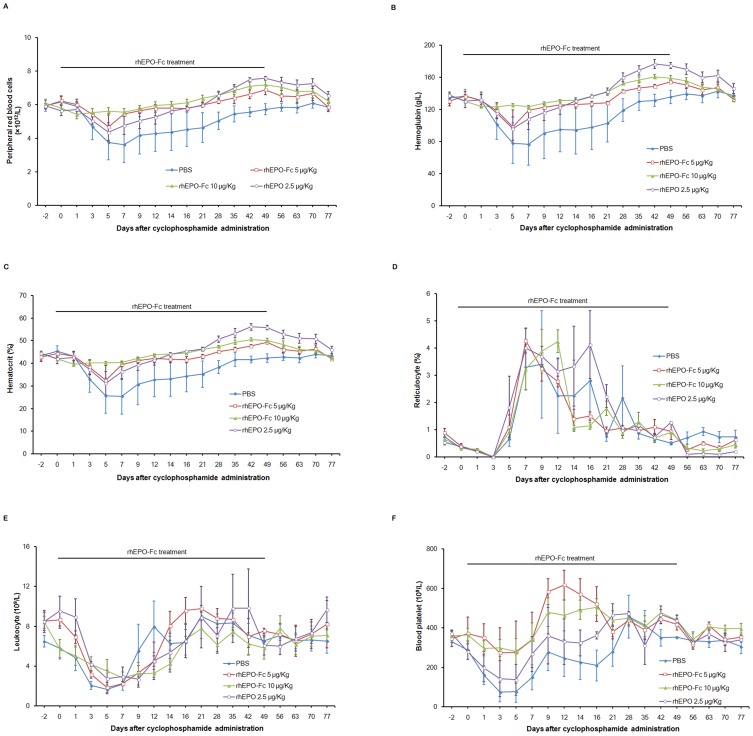
rhEPO-Fc attenuated anemia induced by cyclophosphamide in rhesus monkeys. Blood samples were collected at 0–49 days during treatments and additional 2 times weekly after the treatment ended. RBC (Fig. 4A), hemoglobin (Fig. 4B), hematocrit (Fig. 4C), reticulocyte (Fig. 4D), leukocyte (Fig. 4E) and blood platelet (Fig. 4F) were determined. Data were presented as Mean±SD.

The *in vivo* erythropoietic effect of rhEPO-Fc was further investigated in rhesus monkeys after anemia induction with cyclophosphamide. As shown in Fig. 4A, the mean RBC count of PBS-treated monkeys decreased to the lowest level (3.6×10^12^/L, 61% of the baseline) on day 7 after cyclophosphamide administration and then recovered slowly to the baseline level. rhEPO-Fc dose-dependently alleviated reduction of RBC counts. On day 7, rhEPO-Fc treatment (5.0, 10.0 µg/kg weekly) increased RBC counts to 5.4×10^12^/L, and 5.6×10^12^/L and then restored RBC counts to the baseline level. The effect of rhEPO-Fc (10.0 µg/kg weekly) was similar to that of rhEPO (2.5 µg/kg, 3 times a week). Similar effects were also observed on hemoglobin and hematocrit upon rhEPO-Fc treatment (Fig. 4B and 4C).

The reticulocyte count reduced to zero on day 3 and increased rapidly to the highest (3.4%) on day 9 and then dropped to baseline level on day 21 in PBS-treated anemic rhesus monkeys. Upon rhEPO-Fc treatment, there was some increase of reticulocytes (4.27% for 5.0 µg/kg on day 7, 4.27% for 10.0 µg/kg on day 12), but no significant difference was observed among the monkeys treated with rhEPO, rhEPO-Fc or PBS (Fig. 4D).

Cyclophosphamide also reduced blood platelet and leukocyte counts, which gradually recovered to the baseline levels in PBS-treated rhesus monkeys. Interestingly, rhEPO-Fc (5.0, 10.0 µg/kg weekly) showed some stimulating effects on platelet and leukocyte recovery. However, no significant difference was observed in blood platelet and leukocyte counts in animals treated with rhEPO, rhEPO-Fc or PBS as shown in Fig. 4E and 4F.

## Discussion

The novel fusion protein, rhEPO-Fc, reported in the present study has its unique advantages. First, no mutation was introduced into the EPO molecule itself. Although the mutation on a disulfide bond of EPO structure has been reported to improve its pharmacokinetics and hematopoietic effects [25], some mutations on EPO could change its erythropoietic activity. For instance, EPO mutant S100E induces a significantly lower hematocrit increase than natural EPO while provides similar protection from progressive photoreceptor degeneration [26], [27]. To maintain its erythropoietic property and to avoid unpredicted risk of immunogenicity in clinical application, no mutation was introduced into EPO in our rhEPO-Fc fusion protein.

Second, the Fc region of modified IgG2 with diminished CDC and ADCC function was linked to the EPO molecule. Fusion proteins consisting of the Fc fragment of human IgG have been shown to have significantly longer *in vivo* half-lives while retaining their biological and therapeutic properties [20], [28]. Several fusion proteins comprising an Fc fragment have been successfully developed for clinical application and approved by FDA for treatment of rheumatoid arthritis and chronic plaque psoriasis [29], [30]. In this study, the Fc region of IgG2 was selected to link to the EPO molecule for the following reasons. The IgG2 molecule does not bind to Fc_γ_R, which can activate antibody dependent cellular cytotoxicity (ADCC). In addition, IgG2 is particularly resistant to proteases, which might reduce the clearance of the fusion protein mediated by autoantibody [31]. Furthermore, a site mutation (Pro331Ser mutation) had been made near the carboxyl-terminus of the CH2 domain of human IgG that appears to be important for both FcγR and C1q binding. So the Fc variant should have less complement-activating activity than the natural Fc fragment while remain as a non-binder to FcγR [19]. Thus the novel rhEPO-Fc molecule was expected to have a extended half-life for *in vivo* applications.

In this study, the half-life of rhEPO-Fc were29.5 to 38.9 h at doses of 8, 25, 80 µg/kg in rhesus monkeys and 35.5 to 43.5 h at doses of 16, 50, 160 µg/kg in rats. Compared with the reported pharmacokinetics data, the half-life of the EPO-Fc fusion protein was obviously extended [14]. Administration of rhEPO-Fc once a week exerted similar hematopoietic effects to that of rhEPO given 3 times a week in anemia animal models induced by irradiation, partial renal ablation and cyclophosphamide, which verified the possibly extended half-life of rhEPO-Fc. When repeated subcutaneous injections were performed in rhesus monkeys, no EPO protein was detected before the 2^nd^, 3^rd^ and 4^th^ injections. This indicated that no rhEPO-Fc accumulation in long-term administration. It also supports safe long-term use of rhEPO-Fc as has been reported previously [32].

Reticulocyte is the newly-produced red blood cell and the count is used to assess bone marrow response to an anemic state. Reticulocyte production increases in response to loss of red blood cells. It increases within 2–3 days of a major acute hemorrhage and reaches its peak in 6–10 days [33], [34]. In this study, similar responses of reticulocyte in different anemia models were also observed, and the rhEPO-Fc administration clearly enhanced production of reticulocytes, supporting rapid and efficient recovery of anemia induced by irradiation, cyclophosphamide and partial renal ablation.

In summary, the low administration frequency of rhEPO-Fc (once a week) was shown to have similar erythropoietic effects in a variety of rodent and primate anemia models when compared with rhEPO administrated three times a week. The pharmacokinetics and pharmacodynamics profiles of rhEPO-Fc strongly indicate that rhEPO-Fc could be potentially used as clinical treatment for anemia associated with chronic renal failure or chemotherapy, and future clinical trials are warranted.

## References

[1] Macdougall IC (2012) New anemia therapies: translating novel strategies from bench to bedside. Am J Kidney Dis 59: 444–451. 22192713 10.1053/j.ajkd.2011.11.013

[2] Fukuma S, Yamaguchi T, Hashimoto S, Nakai S, Iseki K, et al (2012) Erythropoiesis-stimulating agent responsiveness and mortality in hemodialysis patients: results from a cohort study from the dialysis registry in Japan. Am J Kidney Dis 59: 108–116. 21890255 10.1053/j.ajkd.2011.07.014

[3] Silva M, Grillot D, Benito A, Richard C, Nuñez G, et al (1996) Erythropoietin can promote erythroid progenitor survival by repressing apoptosis through Bcl-XL and Bcl-2. Blood 88: 1576–1582. 8781412

[4] Eschbach JW, Egrie JC, Downing MR, Browne JK, Adamson JW (1987) Correction of the anemia of end-stage renal disease with recombinant human erythropoietin. Results of a combined phase I and II clinical trial. N Engl J Med 316: 73–78. 3537801 10.1056/NEJM198701083160203

[5] Abels RI (1992) Use of recombinant human erythropoietin in the treatment of anemia in patients who have cancer. Semin Oncol 19 (3 Suppl 8)29–35. 1615337

[6] Henry DH, Beall GN, Benson CA, Carey J, Cone LA, et al (1992) Recombinant human erythropoietin in the treatment of anemia associated with human immunodeficiency virus (HIV) infection and zidovudine therapy. Overview of four clinical trials. Ann Intern Med 117: 739–748. 1416576 10.7326/0003-4819-117-9-739

[7] Sharples EJ, Thiemermann C, Yaqoob MM (2006) Novel applications of recombinant erythropoietin. Curr Opin Pharmacol 6: 184–189. 16483842 10.1016/j.coph.2006.01.003

[8] Joyeux-Faure M (2007) Cellular protection by erythropoietin: new therapeutic implications? J Pharmacol Exp Ther 323: 759–762. 17717190 10.1124/jpet.107.127357

[9] Macdougall IC (2001) An overview of the efficacy and safety of novel erythropoiesis stimulating protein (NESP). Nephrol Dial Transplant (Suppl 3): 4–21. 10.1093/ndt/16.suppl_3.14 11402086

[10] Joy MS (2002) Darbepoetin-alfa: a novel erythropoiesis-stimulating protein. Ann Pharmacother 36: 1183–1192. 12086553 10.1345/aph.1A416

[11] Elliott S, Lorenzini T, Asher S, Aoki K, Brankow D, et al (2003) Enhancement of therapeutic protein in vivo activities through glycoengineering. Nat Biotechnol 21: 414–421. 12612588 10.1038/nbt799

[12] Elliott S, Egrie J, Browne J, Lorenzini T, Busse L, et al (2004) Control of rHuEPO biological activity: the role of carbohydrate. Exp Hematol 32: 1146–1155. 15588939 10.1016/j.exphem.2004.08.004

[13] Jolling K, Ruixo JJ, Hemeryck A, Piotrovskij V, Greway T (2004) Population pharmacokinetic analysis of PEGylated human erythropoietin in rats. J Pharm Sci 93: 3027–3038. 15503315 10.1002/jps.20200

[14] Wang YJ, Hao SJ, Liu YD, Hu T, Zhang GF, et al (2010) PEGylation markedly enhances the in vivo potency of recombinant human non-glycosylated erythropoietin: a comparison with glycosylated erythropoietin. J Control Release 145: 306–313. 20427020 10.1016/j.jconrel.2010.04.021

[15] Dalle B, Henri A, Rouyer-Fessard P, Bettan M, Scherman D, et al (2001) Dimeric erythropoietin fusion protein with enhanced erythropoietic activity in vitro and in vivo. Blood 97: 3776–3782. 11389016 10.1182/blood.v97.12.3776

[16] Kochendoerfer GG, Chen SY, Mao F, Cressman S, Traviglia S, et al (2003) Design and chemical synthesis of a homogeneous polymer-modified erythropoiesis protein. Science 299: 884–887. 12574628 10.1126/science.1079085

[17] Sytkowski AJ, Lunn ED, Davis KL, Feldman L, Siekman S (1998) Human erythropoietin dimmers with markedly enhanced in vivo activity. Proc Natl Acad Sci U S A 95: 1184–1188. 9448306 10.1073/pnas.95.3.1184 PMC18713

[18] Sytkowski AJ, Lunn ED, Risinger MA, Davis KL (1999) An erythropoietin fusion protein comprised of identical repeating domains exhibits enhanced biological properties. J Biol Chem 274: 24773–24778. 10455149 10.1074/jbc.274.35.24773

[19] Sun Lee-Hwei K, Sun Bill NC, and Sun Cecily RY, Fc fusion proteins of human erythropoietin with increased biological activities. Unite states patent. No.6900292B2

[20] Yang JJ, Zhang QD, Zhou J, Gong Z, Tao Q, et al (2007) High Expression, Purification, Quality Control of rhEPO-Fc Fusion Protein,China Biotechnol. 27: 6–9.

[21] Stüben G, Pöttgen C, Knühmann K, Schmidt K, Stuschke M, et al (2003) Erythropoietin restores the anemia-induced reduction in radiosensitivity of experimental human tumors in nude mice. Int J Radiat Oncol Biol Phys 55: 1358–1362. 12654448 10.1016/s0360-3016(03)00012-9

[22] Amann K, Törnig J, Buzello M, Kuhlmann A, Gross ML, et al (2002) Effect of antioxidant therapy with dl-alpha-tocopherol on cardiovascular structure in experimental renal failure. Kidney Int 62: 877–884. 12164869 10.1046/j.1523-1755.2002.00518.x

[23] Shanafelt TD, Lin T, Geyer SM, Zent CS, Leung N, et al (2007) Pentostatin, cyclophosphamide, and rituximab regimen in older patients with chronic lymphocytic leukemia. Cancer 109: 2291–2298. 17514743 10.1002/cncr.22662

[24] Nelius T, Klatte T, de Riese W, Haynes A, Filleur S (2010) Clinical outcome of patients with docetaxel-resistant hormone-refractory prostate cancer treated with second-line cyclophosphamide-based metronomic chemotherapy. Med Oncol 27: 363–367. 19365737 10.1007/s12032-009-9218-8

[25] Way JC, Lauder S, Brunkhorst B, Kong SM, Qi A, et al (2005) Improvement of Fc-erythropoietin structure and pharmacokinetics by modification at a disulfide bond. Protein Eng Des Sel 18: 111–118. 15820978 10.1093/protein/gzi021

[26] Colella P, Iodice C, Di Vicino U, Annunziata I, Surace EM, et al (2011) Non-erythropoietic erythropoietin derivatives protect from light-induced and genetic photoreceptor degeneration. Hum Mol Genet 20: 2251–2262. 21421996 10.1093/hmg/ddr115 PMC3090200

[27] Sullivan TA, Geisert EE, Templeton JP, Rex TS (2012) Dose-dependent treatment of optic nerve crush by exogenous systemic mutant erythropoietin. Exp Eye Res 96: 36–41. 22306016 10.1016/j.exer.2012.01.006 PMC3350094

[28] Xu W, Jones M, Liu B, Zhu X, Johnson CB, et al (2013) Efficacy and Mechanism-of-Action of a Novel Superagonist Interleukin-15: Interleukin-15 Receptor αSu/Fc Fusion Complex in Syngeneic Murine Models of Multiple Myeloma. Cancer Res 73: 3075–3086. 23644531 10.1158/0008-5472.CAN-12-2357 PMC3914673

[29] Goldenberg MM (1999) Etanercept, a novel drug for the treatment of patients with severe, active rheumatoid arthritis. Clin Ther 21: 75–87. 10090426 10.1016/S0149-2918(00)88269-7

[30] Wong VK, Lebwohl M (2003) The use of alefacept in the treatment of psoriasis. Skin Therapy Lett 8:: 1–2, 7. 14610613

[31] Brezski RJ, Oberholtzer A, Strake B, Jordan RE (2011) The in vitro resistance of IgG2 to proteolytic attack concurs with a comparative paucity of autoantibodies against peptide analogs of the IgG2 hinge. MAbs 3: 558–567. 22123056 10.4161/mabs.3.6.18119 PMC3242842

[32] Zhang X, Liu B, Wang L, Cen XB (2007) Long-term toxic effect and immunogenicity of recombinant EPO-Fc in rhesus monkeys. Chinese J New Drugs 16: 196–199.

[33] Adamson JW, Longo DL (2001) Anemia and polycythemia. in: Braunwald E, Fauci AS, Kasper DL, Hauser SL, Longo DL, Jameson JL, ed. New York, pp. 348–354.

[34] Hoffbrand AV, Moss PAH, Pettit JE (2001) Essential Haematology. Fourth Ed., Blackwell Science, Oxford.

